# Novel Henipavirus, Salt Gully Virus, Isolated from Pteropid Bats, Australia

**DOI:** 10.3201/eid3109.250470

**Published:** 2025-09

**Authors:** Jennifer Barr, Sarah Caruso, Sarah J. Edwards, Shawn Todd, Ina Smith, Mary Tachedjian, Gary Crameri, Lin-Fa Wang, Glenn A. Marsh

**Affiliations:** Author affiliations: Commonwealth Scientific and Industrial Research Organisation, Health and Biosecurity, Geelong, Victoria, Australia (J. Barr, S. Caruso, S.J. Edwards, S. Todd, I. Smith, M. Tachedjian, G. Crameri, G.A. Marsh); Duke University-National University of Singapore Medical School, Singapore (L-F. Wang)

**Keywords:** Henipavirus, Salt Gully virus, viruses, zoonoses, bats, Australia, ephrin

## Abstract

We describe isolation and characterization of a novel henipavirus, designated Salt Gully virus, from the urine of pteropid bats in Australia. We noted the virus to be most closely related to Angavokely virus, not reliant on ephrin receptors for cell entry, and of unknown risk for human disease.

Bats of the genus *Pteropus* are natural reservoirs for zoonotic viruses, including the henipaviruses: enveloped, nonsegmented, negative-sense RNA viruses belonging to the family Paramyxoviridae (*1*). Hendra virus (HeV) and Nipah virus (NiV) represent the prototype henipaviruses and have caused fatal zoonotic spillover events from pteropid bats into animals and humans (*2*,*3*). High virulence, broad species tropism, and a lack of approved human vaccines and therapeutics classifies HeV and NiV as risk group 4 pathogens, restricting handling to Biosafety Level 4 (*4*).

Researchers first identified henipaviruses when an outbreak of HeV caused the death of 14 horses and 1 horse trainer in 1994 in Brisbane, Queensland, Australia (*2*). In total, 4 humans and >100 horses have died from HeV infection (*5*,*6*). In 2009, researchers isolated the first nonpathogenic henipavirus, Cedar virus (CedV), from pteropid bat urine collected during bat surveillance activities in Cedar Grove, Queensland, Australia (*7*).

Pteropid bats are the only natural reservoir identified within Australia for henipaviruses and, globally, detection of henipavirus relates most predominantly with pteropid bats. However, reports have noted an increasing number of novel henipa-like viruses detected in species of rodents, shrews, and opossums (*8*–*10*); such viruses have been classified by the International Committee on Taxonomy of Viruses in a new genus, *Parahenipavirus* (*11*).

We describe isolation and in vitro characterization of a novel pteropid bat–borne henipavirus in Australia. We obtained full-length sequences and assessed the virus’s genetic relationship to other henipaviruses. We also compared the virus’s growth, species tropism, and host cell receptor usage with HeV and CedV.

## The Study

On July 11, 2011, we collected 30 pooled bat urine samples from a pteropid bat roost at Bicentennial Park, Boonah, Queensland, Australia, for an HeV surveillance project. We screened samples using quantitative real-time PCR for HeV. Ten samples were negative for HeV and inoculated onto Vero (African green monkey kidney) and primary *Pteropus alecto* kidney (PaKi) cell monolayers. We observed no viral cytopathic effects (CPE) after 7 days; however, the Vero tissue culture supernatant (TCSN) from 1 sample (BO13) tested positive when we employed generic reverse transcription PCR primers for paramyxovirus and henipavirus/morbillivirus (*12*). Sequencing of PCR products revealed a novel henipavirus sequence. Further passage of this virus in Vero cells yielded no CPE, and the virus did not replicate. However, when we inoculated TCSN from Vero cells onto PaKi cells, we observed replication and CPE. We then propagated a working stock in Vero cells, resulting in viral CPE demonstrating small syncytia, cell fusion, and rounded up cells (Appendix Figure). We designated the novel virus Salt Gully virus (SGV) based on the collection location.

**Figure 1:**
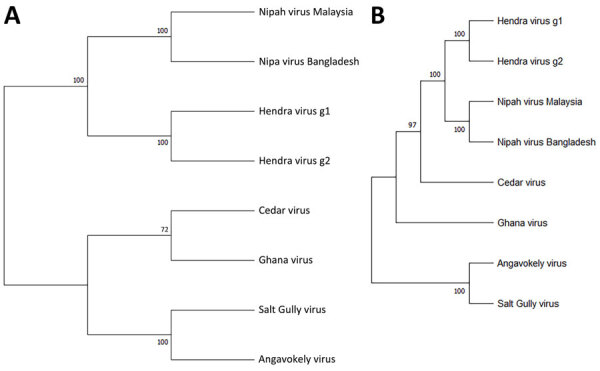
Phylogenetic analysis of members of the genus *Henipavirus* from a study investigating a novel henipavirus, Salt Gully virus, isolated from pteropid bats, Australia**.** A) We aligned complete L protein amino acid sequences by using ClustalW (https://www.genome.jp/tools-bin/clustalw) and inferred evolutionary history by using the maximum-likelihood method and the Jones-Taylor-Thornton matrix-based model. B) We aligned complete virus genome sequences by Muscle software and inferred evolutionary history by using the maximum-likelihood method and general time reversible plus gamma plus invariate sites model. Bootstrap support values (1,000 replicates) are shown next to each branching node. Evolutionary analyses were conducted in MEGA11 (https://www.megasoftware.net).

Next-generation sequencing of RNA extracted from TCSN on the Illumina platform (https://www.illumina.com), followed by genome assembly and analysis, revealed a large, complete genome of 19,884 nt (GenBank accession no. PV233879), adhering to the rule of 6 for paramyxoviruses (*13*). This genome included 6 distinct open reading frames that encoded 6 proteins: nucleocapsid (N), phosphoprotein (P), matrix (M), fusion (F), glycoprotein (G), and RNA polymerase (L). In addition, an alternative start codon within the P gene indicated the presence of a 7th open reading frame and a C protein, consistent with other henipavirus genomes. Whole-genome nucleotide alignment with other known henipaviruses showed that SGV shared 38% identity with HeV and NiV, 37% identity with Angavokely virus (AngV), and 35% identity with CedV and Ghana virus. We determined SGV to be phylogenetically most closely related to AngV (Figure 1).

**Figure 2:**
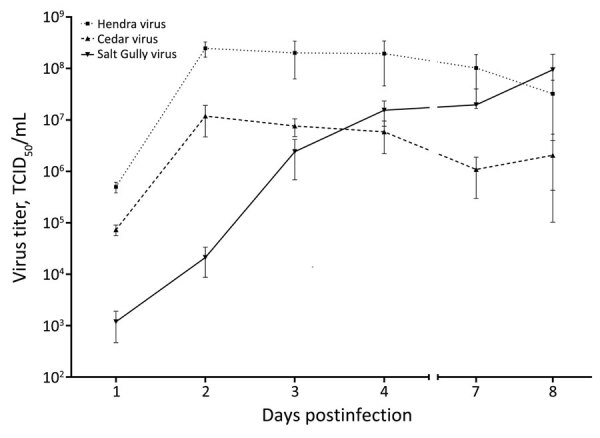
Growth of Salt Gully virus in Vero cells compared with Hendra virus and Cedar virus from study investigating a novel henipavirus, Salt Gully virus, isolated from pteropid bats, Australia**.** Vero cell monolayers were infected with each virus at a multiplicity of infection of 0.01 in triplicate, and tissue culture supernatant was collected until day 8 for TCID_50_ assay to determine the viral titer. Error bars indicate the standard deviation of the mean between replicates. TCID_50_, 50% tissue culture infectious dose.

Investigating species tropism and growth kinetics, we found that SGV infected Vero, pteropid bat, and human cell lines, showing varying levels of CPE by 3 dpi. We observed no viral CPE in porcine or primary equine cell lines by 7 dpi. HeV replicated in all 5 cell lines, and CedV replicated in all except equine (Table). Initially, SGV displayed delayed replication in Vero cells; however, SGV reached maximum viral titer by 8 dpi at a titer comparable to HeV and CedV, which peaked by 2 dpi and then declined (Figure 2).

**Table:** Growth of viruses in 5 mammalian cell lines 3 days postinfection in study of novel henipavirus, Salt Gully virus, isolated from pteropid bats, Australia*

Virus	% Cell monolayer infected				
	Vero	PaKi	HeLa	EFK	PK15a
Salt Gully virus	20−50	20−50	<20	0	0
Cedar virus	>50	>50	>50	0	>50
Hendra virus	100	20−50	100	20−50	<20

*Cell types: Vero, African green monkey kidney; PaKi, Pteropus alecto kidney; HeLa, human epithelial; EFK, equine fetal kidney; PK15a, porcine kidney.

**Figure 3:**
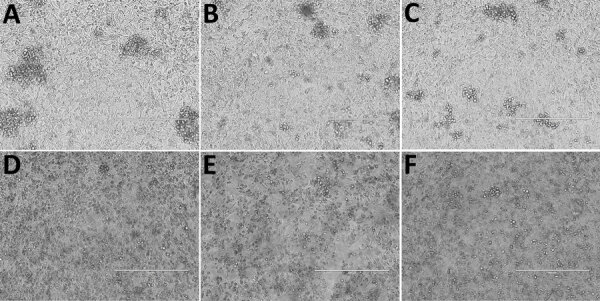
Ephrin-B2 and ephrin-B3 host cell receptor usage from a study investigating a novel henipavirus, Salt Gully virus (SGV), isolated from pteropid bats, Australia. Three cell lines—HeLa-USU, recombinant HeLa-USU expressing ephrin-B2, and HeLa-USU expressing ephrin-B3—were inoculated with SGV and observed daily for viral cytopathic effects (CPE). CPE was seen in all 3 cell lines by 5 dpi, indicating that SGV does not use ephrin-B2 or ephrin-B3 as a host cell receptor. A) Uninfected HeLa-USU cells; B) uninfected HeLa-USU cells expressing ephrin-B2; C) uninfected HeLa-USU cells expressing ephrin-B3; D) SGV-infected HeLa-USU cells; E) SGV-infected HeLa-USU ephrin-B2 cells; and F) SGV-infected HeLa-USU ephrin-B3 cells. Scale bars indicate 400um.

We used human epithelial (HeLa)-USU cells and recombinant HeLa-USU cell lines expressing ephrin-B2 or ephrin-B3 to assess SGV receptor usage. We observed viral CPE in HeLa cells expressing ephrin-B2 and ephrin-B3 for HeV and in ephrin-B2-expressing HeLa cells for CedV after 2 dpi. HeV and CedV did not infect HeLa-USU cells, as shown previously (*14*). In contrast, SGV caused CPE in all 3 HeLa cell lines after 5 dpi, indicating usage of an unknown receptor that is neither ephrin-B2 nor ephrin-B3 (Figure 3).

## Conclusions

We detected SGV in bat urine samples collected in Australia in 2011 and successfully isolated the virus using pteropid bat cell lines. Initially, the CPE of inoculated Vero cells was minimal, requiring passage in pteropid kidney cells to achieve efficient virus replication. Despite the identification of multiple new henipa-like viruses in pteropid bats and small mammals globally, viral isolation is largely unsuccessful and remains a technical challenge. In our study, employing primary cell lines derived from the relevant species resulted in virus isolation.

Full-length genome sequencing of SGV revealed a genome organization consistent with other known henipaviruses, with all predicted henipavirus protein ORFs identified. Whole-genome alignments comparing SGV to other henipaviruses revealed 35%–38% identity at a nucleotide level. Of interest, phylogenetic analysis of the genome clusters SGV with AngV, a henipavirus that was detected in fruit bats (*Eidolon dupreanum*) in Madagascar in 2022 (*15*).

We assessed the ability for SGV to infect various mammalian cell lines in vitro, including Vero, PaKi, HeLa, equine fetal kidney, and porcine kidney cells. SGV’s notable ability to infect human cells underscores its potential for human infection. Unlike HeV, which infected all 5 cell lines, SGV did not cause CPE in porcine or equine cells. SGV could grow to high titer in Vero cells, albeit slower than HeV. Collectively, these results indicate SGV may not have the broad species tropism of pathogenic henipaviruses but could pose a human risk.

Ephrin-B2 and ephrin-B3 are host cell receptors for HeV and NiV (*14*), and the sequence conservation of those ligands across many species supports the broad species tropism of classical henipaviruses. The HeLa-USU cell line we used has been shown to lack expression of ephrin-B2 and ephrin-B3 and was used to determine the functional host cell receptor for CedV. In our study, SGV infected all 3 HeLa-USU cell lines, demonstrating that SGV cell entry is not reliant on ephrin-B2 or ephrin-B3, suggesting SGV uses a yet unidentified host cell receptor. In comparison, research has shown the glycoprotein G of Ghana virus could bind to ephrin-B2 (but not ephrin-B3), whereas predicted structure-based alignments suggest AngV is unlikely to use ephrin receptors for host cell entry (*15*). Further characterization is required to determine the functional host cell receptor for SGV and accurately assess pathogenicity in other species.

In summary, we identified, isolated, and characterized a novel henipavirus from pteropid bats in Australia. Amid the increasing discovery of novel henipa-like viruses in new locations and species, SGV is a true henipavirus and phylogenetically clusters with other bat henipaviruses. However, this virus’s pathogenicity remains unknown, making the susceptibility of human and animal populations in Australia uncertain.

Appendix Additional information for novel henipavirus, Salt Gully virus, isolated from pteropid bats, Australia
