# Supplementary material for: Novel Henipavirus, Salt Gully Virus, Isolated from Pteropid Bats, Australia
Source: Emerg Infect Dis. 2025 Sep;31(9):1824–8. doi: 10.3201/eid3109.250470 (PMC12407197; doi:10.3201/eid3109.250470)
Supplement: Appendix — Additional information for novel henipavirus, Salt Gully virus, isolated from pteropid bats, Australia [file 25-0470-Techapp-s1.pdf]

Article DOI: http://doi.org/10.3201/eid3109.250470

EID cannot ensure accessibility for supplementary materials supplied by authors. Readers who have difficulty accessing supplementary content should contact the authors for assistance.

Novel Henipavirus, Salt Gully Virus, Isolated from Pteropid Bats, Australia

Appendix

Materials and Methods

Ethics statement

Bat urine sample collection was approved by the (then) Queensland Department of Employment, Economic Development and Innovation Animal Ethics Committee and conducted under Permit SA 2011/12/375 and the Queensland Department of Environment and Resource Management Scientific Purposes Permit WISP05810609.

Virus isolation

Pooled bat urine was collected from beneath a pteropid bat roost located at Bicentennial Park, Boonah, Qld on 11th July 2011 as part of a wider HeV surveillance project. Urine was collected, transported, and stored as previously described (*1*). In the laboratory, urine samples were thawed, centrifuged, diluted, and re-centrifuged before inoculation on Vero and primary *Pteropus alecto* kidney (PaKi) cells, as previously described (*1*).

Genome analysis and characterization

Viral RNA was extracted from TCSN using a MagMAX-96 Viral RNA Isolation Kit (Applied Biosystems) following the manufacturer's instructions. The isolated virus was initially characterized using a hemi-nested PCR with degenerate primers following the protocols described previously (*2*), followed by Sanger sequencing of the PCR products.

To sequence the whole genome, 100 µL virus-containing supernatant was added to 300 µL of TRIzol reagent and RNA extracted using a Direct-zol RNA Miniprep kit (Zymo), including an in column DNaseI digestion. A REPLI-g WTA Single Cell kit (Qiagen) was used for isothermal amplification, followed by processing with a Genomic DNA and Concentrator 10

kit (Zymo). Fragmentation and dual-index library preparation were conducted using Nextera XT DNA Library Preparation kit (Illumina), and denatured libraries were sequenced using a MiniSeq High Output Reagent kit (Illumina). The virus genome was assembled using the SPAdes de novo assembly tool. Full length sequence is available in GenBank (accession number PV233879). Genome analysis, nucleotide and amino acid alignments, and phylogenetic tree construction were performed using Mega11 software (*3*).

Infection of mammalian cell lines

Five mammalian cell lines; Vero (African green monkey; source ATCC CCL-81), PK15a (porcine; source USDA), HeLa (human; source ATCC CCL-2), PaKi (*Pteropus alecto*; made in-house) and EFK (equine; source Melbourne University), were seeded at a concentration of 250,000 cells/well in 6-well plates and incubated overnight at 37°C in a 5% CO_2 incubator. Confluent cell monolayers were infected with SGV, CedV or HeV at an MOI of 0.01 and incubated at 37°C in a 5% CO_2 incubator. Cell monolayers were monitored daily for viral CPE.

Virus replication in Vero cells

Vero cells were seeded at a concentration of 250,000 cells/well in 6-well plates and incubated overnight at 37°C in a 5% CO_2 incubator. In triplicate, cell monolayers were infected with SGV, CedV or HeV at an MOI of 0.01 and incubated at 37°C in a 5% CO_2 incubator. TCSN was collected daily from all wells for the following time points: 1-, 2-, 3-, 4-, 7- and 8-days post infection, and stored at −80°C for later analysis of virus titer.

Virus titers were determined by $TCID_{50}$ assay in a 96-well plate format using Vero cells, with titers calculated using the Reed-Muench method (*4*).

Infection of recombinant ephrin-B2 and ephrin-B3 cell lines

HeLa-USU cells and recombinant HeLa-USU cell lines expressing ephrin-B2 or ephrin-B3 were prepared previously as described (*5*). The three cell lines were seeded at a concentration of 250,000 cells/well in 6-well plates and incubated overnight at 37°C in a 5% CO_2 incubator. Confluent cell monolayers were infected with SGV, CedV or HeV at an MOI of 0.01 and incubated at 37°C in a 5% CO_2 incubator. Cells were monitored daily for viral CPE.

References

1. Marsh GA, de Jong C, Barr JA, Tachedjian M, Smith C, Middleton D, et al. Cedar virus: a novel Henipavirus isolated from Australian bats. PLoS Pathog. 2012;8:e1002836. PubMed https://doi.org/10.1371/journal.ppat.1002836

2. Tong S, Chern SWW, Li Y, Pallansch MA, Anderson LJ. Sensitive and broadly reactive reverse transcription-PCR assays to detect novel paramyxoviruses. J Clin Microbiol. 2008;46:2652–8. PubMed https://doi.org/10.1128/JCM.00192-08

3. Tamura K, Stecher G, Kumar S. MEGA11: Molecular evolutionary genetics analysis version 11. Mol Biol Evol. 2021;38:3022–7. PubMed https://doi.org/10.1093/molbev/msab120

4. Reed LJ, Muench H. A simple method of estimating fifty percent endpoints. Am J Epidemiol. 1938;27:493–7. https://doi.org/10.1093/oxfordjournals.aje.a118408

5. Bonaparte MI, Dimitrov AS, Bossart KN, Crameri G, Mungall BA, Bishop KA, et al. Ephrin-B2 ligand is a functional receptor for Hendra virus and Nipah virus. Proc Natl Acad Sci U S A. 2005;102:10652–7. PubMed https://doi.org/10.1073/pnas.0504887102

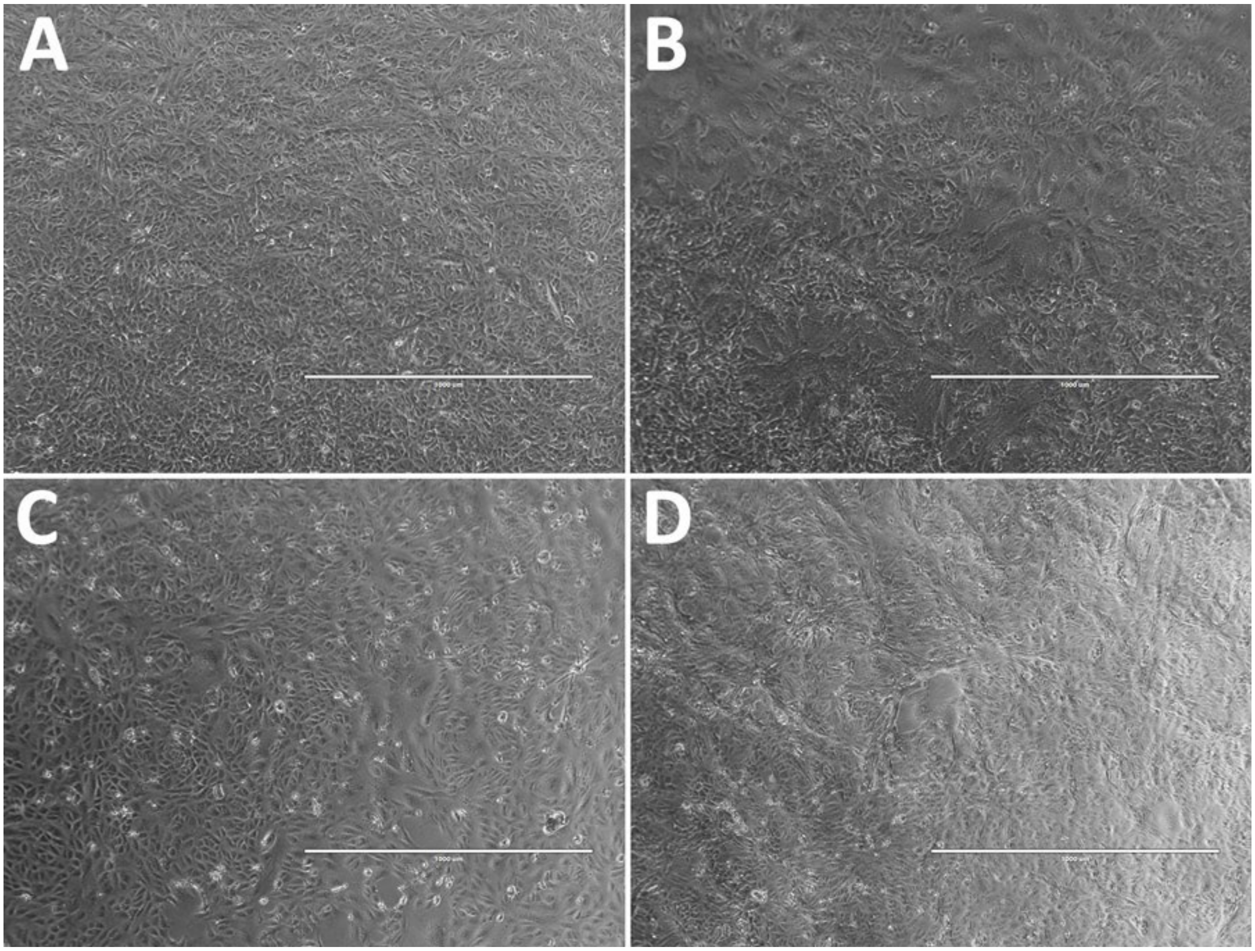

Appendix Figure. Comparison of viral cytopathic effects observed in Vero cells from a study of a novel henipavirus, Salt Gully virus (SGV), isolated from pteropid bats, Australia. Vero cells were infected with Hendra virus (HeV), Cedar virus (CedV), and SGV at a multiplicity of infection of 0.01. (A) Uninfected cells 24 h postinfection, (B) HeV-infected cells 24 h postinfection, (C) CedV-infected cells 72 h postinfection, and (D) SGV-infected cells 72 h postinfection. Photos taken at 4x magnification.
